# The Dilemmas of Scientific Research Cooperation and Their Resolution From the Perspective of Evolutionary Psychology

**DOI:** 10.3389/fpsyg.2019.02561

**Published:** 2019-11-15

**Authors:** Gaofeng Wang, Qingqing Kong

**Affiliations:** Department of Philosophy of Science and Technology, School of Humanities and Social Sciences, University of Science and Technology of China, Hefei, China

**Keywords:** research cooperation, evolutionary psychology, problem, strategy, method

## Abstract

Scientific research cooperation has become a mainstream trend of social development. It can promote resource sharing, help group members complement each other’s advantages, and improve scientific research efficiency. With the deepening of scientific research cooperation, there have also been problems such as the uneven strength of partners, gender discrimination, and group exclusionary behavior. People often explore the causes of these problems in terms of the process of scientific research cooperation, but doing so fails to solve the substantive problems effectively. We thus seek to trace the psychology of people participating in scientific research cooperation from the perspective of evolutionary psychology so as to analyze the root causes of scientific research cooperation problems. This paper first discusses the importance of scientific research cooperation, then enumerates common problems in scientific research cooperation, analyzes them from the perspective of evolutionary psychology, and proposes solutions to these problems from the perspective of regulating people’s psychology. This article illustrates how the many perspectives and theories of evolutionary psychology can solve problems in other disciplines and fields, and indeed that all human social activities can be explained by evolutionary psychology, which opens up a broader field of research for evolutionary psychology.

## Introduction: The Importance of Scientific Research Cooperation

Since the birth of human society, cooperation has accompanied human development. At first, human beings were forced to cooperate in order to survive, which allowed them to avoid the threat of death and gain opportunities for survival and reproduction (Cohen and Bernard, 2013). With the technological revolution, human needs have also shifted from basic survival needs to emerging technology needs. Emerging disciplines are interconnected, and related research projects have encountered problems such as difficult goals, large investment, and lack of talent (Xie and Liu, 2006). Under these circumstances, scientific research cooperation is an effective way for the parties to share resources, complement each other’s advantages, and improve scientific research efficiency. In an early work on the sociology of science, Merton pointed out that the development of science will gradually merge with the needs of society, mainly through scientific research exchange, competition, and cooperation. Scientific research cooperation will become an important driving force for the development of scientific productivity as well as an inevitable trend of social development.

## Problems in Scientific Research Cooperation

Despite the many benefits of scientific research cooperation, there have been many problems with the deepening of scientific research cooperation. Existing research on scientific research cooperation points out that common problems in scientific research cooperation include unsatisfactory expected returns (Mason, 1989), cultural language barriers (Cohen, 2001), spatial distance barriers (Zeng et al., 2019), scientific research partners, the risk of betrayal of uneven strength (Zhao and Liao, 2013), the unequal opportunities for men and women in scientific research cooperation (Bozeman and Gaughan, 2011), the identification of internal groups, the exclusion of external groups in scientific research cooperation (Eaton et al., 2011), and so on. This article is based on the perspective of evolutionary psychology; therefore, the issue of scientific research cooperation caused by individual psychological motives is taken as an example to explore the psychological mechanism and corresponding solutions of cooperators.

### The Problem of the Unequal Status of Collaborators in Research Cooperation

The essence of scientific research cooperation is sharing resources for mutual profit, but as the strength of the two parties is not certain and there must be strengths and weaknesses in their capabilities, resources, and positions, this leads to the unequal status of the research parties. In scientific cooperation between parties with obvious strengths and weaknesses, two situations will occur: strong partners are more inclined to compete, on the one hand, to maintain their strong position and, on the other, to acquire more resources for greater benefits (Avrahami et al., 2014). The weak partners tend to cooperate, pursue equality with their partners, and protect their legitimate interests. Two different ideologies cause conflicts in cooperation (Kimbrough et al., 2014).

### Gender Discrimination in Research Cooperation

With the rise of the women’s liberation movement, increasing numbers of women have entered the academic labor market; the phenomenon of inequality between men and women seems to have been alleviated, but the gap between men and women in academic status is still quite obvious. Especially in science and engineering, the situation of female academic workers presents a “leaky pipeline” phenomenon (the “leaky pipeline” refers to the loss of talent in the middle of an academic career; Bilimoria et al., 2008). Studies have shown that men generally cooperate with and give more opportunities to other men (Bozeman and Corley, 2004). The view of men as being more adept at academics than women has been deeply ingrained, forcing women to be passive in scientific cooperation, which has led to imbalance gender ratios in research cooperation circles.

### Intra-group Identity and Extra-group Exclusionary Behavior in Scientific Research Cooperation

With the deepening of scientific research cooperation, cooperation between different regions, disciplines, and groups has become increasingly widespread. In the fusion of different individuals with each other and with groups, contradictions and conflicts are inevitable. Individuals have different perspectives on their own groups and collaborators due to individual cultural differences (Song et al., 2018). When a foreign group appears, there will be preferences for those within the group: members will show greater fondness, more trust, and greater partiality to their group (Hewstone et al., 2002). Studies have shown that when a group works together, the members of the group are usually classified, calling the members of one’s own group “we” and the other side of the partnership “they” (Eaton et al., 2011). An obvious dividing line has been formed within the group, hindering scientific research cooperation from being carried out in a harmonious atmosphere, which also directly affects the progress of scientific research cooperation.

## Strategies for Solution

From the beginning of biological evolution, cooperation exists between different species. The difficult living environment forces individuals to realize that only seeking cooperation can survive. This consciousness is an evolution of human psychology and a common scene of complex cognitive ability in human evolution. This in turn promotes extensive cooperation (Tomasello, 1999). Human psychology also produces altruism (Lehmann and Keller, 2006), unequal psychology (Wilkinson and Pickett, 2017), selfishness, and so on (Brewer and Caporael, 1990), so cooperation with various psychological evolution also faces new problems. Exploring the psychological mechanism of the problem is the key to solving the problem of scientific research cooperation.

### Evolutionary Psychological Causes of Scientific Cooperation

Unfairness arising from status inequality in scientific research cooperation may stem from dominance in the field of evolutionary psychology (Trivers, 1971). The strong can maintain their dominance by virtue of existing resources and can have more resources and survival opportunities, while the weak in a passive position, without active choice, can only obey the distribution of the strong, dependent on the supply of the strong, in a state of being dominated. Natural selection tends to favor the dominant individual and evolve it.

The first is the physiological relationship between the mother and the child due to the kinship (Antfolk et al., 2017). For example, you can be sure that the child you gave birth is a biological child. The intimacy is highly certain, and the certainty of the child's relationship with the father and all other relatives needs to be tested. However, the second reason for father and child is that women tend to have more chances to choose a spouse than men (Anderson et al., 2007). For example, if a man has children and can have a large number of sexual partners to get more children, but women cannot increase the number of offspring by more men, so women are more willing to invest more time in raising children and families.

There are also internal group identity and external group rejection in scientific research cooperation. Evolutionary psychology believes that this is the individual's cultural group identity psychology (Masuda, 2012). This sense of group identity directly affects the efficiency of cooperation (Zuo et al., 2019).

The above outlines the psychological motivations underlying the problems in scientific research cooperation. This paper proposes corresponding coping strategies from the perspective of evolutionary psychology.

### Coping Strategies From the Perspective of Evolutionary Psychology

#### Strengthening Group Awareness and Promoting Cooperation Equality

The reason for the uneven strength of the two sides is that the private interests of the two parties in scientific cooperation are different, but both are in a cooperative community with a common goal of cooperation (Lehmann and Keller, 2006). On the one hand, by strengthening the target awareness of the group, if the strong party pays more attention to the collective interests, this will help the strong side overcome the temptation of personal interests and promote fairness and justice in scientific research cooperation. On the other hand, increasing the social identity of the strong side, gaining more resources by increasing their own visibility, and using reciprocal psychology to stimulate the strong side to play a leading role in cooperation.

#### Establishing a Concept of Academic Equal Rights

The concepts underlying gender discrimination in scientific research cooperation are deeply rooted. In terms of psychology, they are not only due to the differences in the division of labor between men and women but also stem from the unequal investment time in parenting. Throughout human evolution, individuals have expanded their groups by reproducing, but the tasks of reproduction and of rearing future generations are mostly performed by women. Over time, men have gained the resources to survive and to control women. At the same time, the division of labor between men and women also creates a lack of fatherly love for many children, which is not conducive to the healthy growth of their children. We recommend that men invest more time and energy in parental care to make up for the uneven time spent by men and women in family and research.

#### Reclassification Within the Group to Alleviate Rejection

The identification of self-groups and the rejection of other groups in scientific research cooperation are also a kind of prejudice. If reorganization is allowed to disrupt the original interests and relationship of a group through cooperation with another, they will agree with each other to achieve new goals. We propose that in research cooperation, the parties regrouped, the partners classified and reorganized by their different professions, and new targets set for the reorganized groups, and then, as the group is driven by the goal of achieving common goals, the sense of group rejection will be rejected and the purpose of the scientific research cooperation achieved.

## Summary and Outlook

This paper analyzes four common problems in scientific research cooperation from the perspective of evolutionary psychology and proposes corresponding countermeasures. Collaboration with free-riding behaviors with various signatures stems from selfishness and reciprocal altruism, and we suggest that the other collaborators should inform their peers of free-riding behavior partners, reducing their social recognition and opportunities for scientific research cooperation. The phenomenon of unequal cooperation stems from the most primitive level of domination, and the strategy proposed to address this is to turn the psychology of needing social identity to obtain more resources toward affirming the contribution of the strong party and publicizing its contribution to the cooperation. The reason for gender discrimination is the different divisions of labor between men and women. We suggest that men establish academic equality between men and women, on the one hand, and assume the responsibility of fathers to raise their children, on the other hand, and thereby balancing the time and energy spent by men and women in the family. Intra-group identity and external exclusion behavior in scientific research cooperation stem from the sense of group identity. The groups in cooperation can be regrouped to encourage scientific research cooperation with the motive of achieving their new common goals so as to reduce the impact of extra-group exclusion behaviors on scientific research cooperation (Hewstone et al., 2002).

Scientific research cooperation has expanded from individual cooperation to group cooperation, discipline cooperation, industry-university-research cooperation, and even more widely, and the resultant problems are increasing. This article only discusses some of the research cooperation issues. Follow-up research can use the paradigms of other disciplines to analyze the problems of scientific research cooperation from the perspective of evolutionary psychology. For example, scientometrics is a model for cross-disciplinary studies drawing on the advantages of different disciplines (Chen and Qin, 2017). It has been shown that it is difficult to solve the problems in scientific research cooperation from the perspective of only one discipline. It is expected that future research can use the advantages of different disciplines to analyze problems from multiple angles to find the optimal solution in scientific research cooperation.

## Author Contributions

GW conceived and wrote the frame design. GW and QK wrote the manuscript. All authors revised the manuscript.

### Conflict of Interest

The authors declare that the research was conducted in the absence of any commercial or financial relationships that could be construed as a potential conflict of interest.
